# The impact of vaginal cone therapy on stress urinary incontinence compared with transobturator tape

**DOI:** 10.4274/tjod.galenos.2019.89137

**Published:** 2019-10-10

**Authors:** Rıza Dur, İltaç Akkurt, Bora Coşkun, Gamze Dur, Buğra Çoşkun, Mehmet Ünsal, Ahmet Akın Sivaslıoğlu

**Affiliations:** 1 University of Health Siences, Etlik Zübeyde Hanım Maternity and Women Hospital, Clinic of Obstetrics and Gynecology, Ankara, Turkey; 2 Bursa Anadolu Hospital, Clinic of Obstetrics and Gynecology, Bursa, Turkey; 3 Liv Hospital, Clinic of Obstetrics and Gynecology, Ankara, Turkey; 4 Çifteler Stale Hospital, Clinic of Obstetrics and Gynecology, Eskişehir, Turkey; 5 Muğla Sıtkı Koçman University Faculty of Medicine, Department of Obstetrics and Gynecology, Muğla, Turkey

**Keywords:** Stress urinary incontinence, vaginal cone therapy, transobturator tape, conservative treatment

## Abstract

**Objective:**

To emphasize the efficiency of vaginal cone (VC) therapy in stress urinary incontinence (SUI) through a comparison with transobturator tape (TOT).

**Materials and Methods:**

A prospective randomized controlled study was conducted at the Etlik Zübeyde Hanım Maternity and Women Hospital during a one year study period. Forty women were allocated into two equal groups; those treated with VCs for a 3 month period, and women who underwent TOT procedures. These women were followed up at 6 weeks and 6 months after the treatments. Subjective cure was assessed using Wagner’s Quality of Life Questionnaire. Objective cure was evaluated through a cough stress and pad test results.

**Results:**

Maternal demographic features were comparable among groups. We observed improvement in pad weight test among groups when compared with the pretreatment state (p=0.015, p=0.005). Although the subjective cure rate was similar in both groups at the 6^th^ week and 6^th^ month follow up (65% vs. 75%; 75% vs. 80%) (p>0.05), the objective cure rate was significantly higher in the TOT group than in the VC group, as expected (10% vs. 80%; 30% vs. 75%) (p<0.05).

**Conclusion:**

The main treatment of SUI is surgery; however, VC could be offered as an alternative treatment for women who refuse surgery, those at high risk for surgery or it could be used temporarily before surgery.

**PRECIS:** Vaginal cone (VC) should not be considered as one of the alternatives to surgical treatment in stress urinary incontinence but VCs may be considered temporarily or in combination with other surgical procedures.

## Introduction

Stress urinary incontinence (SUI) is an involuntary leakage of urine during an exerted effort, exertion, sneezing, or coughing^(1)^. Approximately 50% of patients diagnosed with urinary incontinence have SUI^(2)^. SUI may occur in 4% to 35% of women^(3)^. SUI is the most common type of urinary incontinence in young women and is more common between the age of 45 and 49 years^(4)^. It is associated with serious economic, social, and psychological problems that affect women’s health^(1)^. There are several treatment options for SUI. Depending on the clinical findings and on the severity of symptoms, SUI can be managed both with conservative methods including pelvic floor exercises, vaginal cones (VC) and general lifestyle modifications, and surgery. It can be treated surgically with procedures such as Burch colposuspension, vaginal slings or tension-free tapes, and injection of bulking agents alongside the urethra. Techniques aimed to strengthen pelvic floor muscles are considered as the first-choice treatment due to the low risk of adverse effects and the low-to-moderate costs^(3)^. Midurethral slings or tapes are widely used as a surgical procedure in SUI, first described by Herbison and Dean^(4)^ in 1995. The rationale of transobturator passage is the restoration of hammock-like support and the avoidance of rare but serious bladder complications such as injury to major vessels or the bowel. Pelvic floor muscle exercises should be encompassed in the first-line of conservative management of women with SUI^(5)^. VC were initially proposed in 1985 by Plevnik^(6)^ to strengthen pelvic floor muscles. Non-surgical treatment of SUI is not the definitive choice; however, it improves the quality of life of women with SUI. The present study aimed to compare the effectiveness of VC therapy and transobturator tape (TOT) in women with SUI, which has not been thoroughly addressed in the literature^(6,7)^.

## Materials and Methods

**Figure 1:**
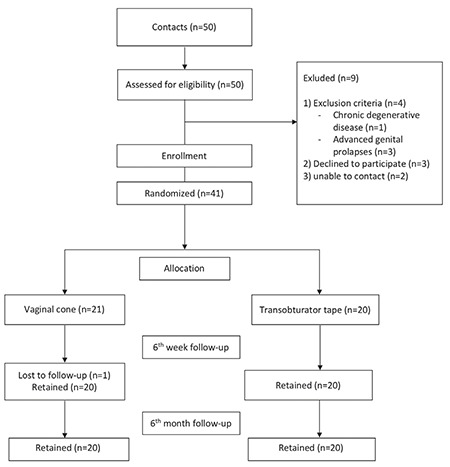
Flow diagram of the study

Women with urodynamic SUI were enrolled in this, controlled, randomized trial at the department of urogynecology and reconstructive pelvic surgery, a division of the gynecology department at a tertiary research and training hospital, Ankara, during a one-year study period. The study was approved by the Hospital’s Institutional Review Board, and informed consent was obtained from each participant. Forty patients were allocated into two groups; patients treated with VC (n=20, group 1) and patients who underwent TOT (n=20, group 2). Women who had urodynamic stress incontinence were confirmed through a positive cough stress test, and >3 g leakage as measured using a pad test with a standardized bladder volume (200 mL) without detrusor overactivity^(8)^. Exclusion criteria were considered as chronic degenerative diseases, advanced genital prolapses, pregnancy, active or recurrent urinary tract infections, vulvovaginitis, atrophic vaginitis, and continence surgery within one year (Figure 1). VC (StepFree VC, SRS Medical, USA) were implemented per the original definition of Plevnik^(9)^, and additionally by a gynecologist at the urogynecology clinic. Patients applied the cones weighted at 20-70 g for 15 minutes twice a day. At the initial visit, patients were guided as to their use. After the first weight was successfully implemented for 15 minutes twice a day, the weight would be prolonged for a week. Afterwards, the VC weight gradually increases and ends within a 3 month treatment period at 70 g. TOT (polypropylene; Dowmedics Co. Ltd., Wonju, Korea) was performed by the same surgeon under spinal anesthesia as described by Delorme^(10)^. Patients were invited for postoperative follow up at six weeks and six months. At each visit, urinary symptoms and other problems were recorded, and a cough stress test was performed, after filling the bladder with 200 mL of water and provoking the patient in a 45° upright sitting position. All patients were also asked to complete the validated Wagner’s Quality of Life Questionnaire before and after treatment as adapted for Turkish women^(11)^. The results are classified as follows: 1-28 points were recorded for minimal incontinence, 29-56 points recorded for moderate incontinence, and 57-84 points for severe incontinence. Treatment outcome was assessed with respect to overall complication and treatment rates. Complications were intraoperative bladder injury, voiding dysfunction lasting more than 1 month after surgery or requiring surgical intervention such as urethral dilation, tape release, or urethrolysis, de novo urgency, recurrent urinary tract infection, and mesh erosion. Women without subjective symptoms of leakage and/or objective leakage on a cough stress test (cure/dry) were defined as completely treated.

### Statistical Analysis

Data were analyzed using the SPSS version 12.0 software package (2004; SPSS Inc., Chicago, IL, USA). Descriptive statistics of the data were determined in the form of mean ± standard deviations and range. Using our data, which represent categorical data, we used the chi-square test to determine the significance of the study with p<0.05 being accepted as significance.

## Results

A total of 40 patients were involved to our study. Table 1 shows the clinical and demographic features of the patients in each group. The mean surgical time and intraoperative blood loss were 14±2 minutes and 35 mL, respectively. There were no intraoperative or postoperative complications in the TOT group. Although we observed a significant decrease in pad weights in the two groups, the TOT group was superior to the VC group at both the 6^th^ week and 6^th^ month follow ups (Table 2). The rate of negative stress test was higher in the TOT group than in the VC group (85% vs. 50% at 6 weeks and 75% vs. 50% at 6 months, respectively) (p<0.05). The results of the quality of life questionnaire are shown in Table 3 and Table 4. There was significant recovery in both groups at the end of the 6^th^ week when compared with pretreatment scores (p<0.05). We observed complete healing in two patients, mild incontinence in 14 patients, and severe incontinence in one women in the TOT group at the 6^th^ week follow-up. By contrast, one patient had no symptoms, mild incontinence was present in nine patients, and severe incontinence was observed in four patients in the VC group. Moreover, we determined complete healing in one patient, mild incontinence in 12 patients, and severe incontinence in two patients at the 6^th^ month follow up in the VC group. On the other hand, 8 patients had no symptoms, seven women had mild incontinence, and three patients had severe incontinence in the TOT group at the 6^th^ month follow up. According to the survey, the subjective cure rate of women in the VC group and TOT group was 65% and 75% at the 6^th^ week follow up, respectively (p>0.05). The objective cure rate in group 1 (n=2) was significantly lower than in group 2 (n=16) at the sixth week follow up (10% vs. 80 %). At the sixth month follow up, the subjective cure rate was similar between the groups (75% vs. 80%) (p>0.05). However, objective cure rates were significantly lower in the VC group (30% vs. 75%) (p<0.05).

## Discussion

Although SUI is not a life-threatening health concern, it affects approximately 200 million women and it influences the patients’ social, psychological, occupational, domestic, physical, and sexual well-being^(1)^. In 1982, Ulmsten hypothesized that urinary continence is related 1/3 of mid-urethra with the highest pressure^(12)^. TOT aims to provide urethral support tissue via a minimally invasive procedure as defined by Delorme in 2001. According to the literature, vaginal slings seem to be the best option in SUI treatment with lower complication rates and better short- and long-term outcomes than other surgical procedures^(13,14,15,16)^. Pelvic floor muscle training (PFMT) is a conservative treatment for SUI, which aims to strengthen pelvic floor muscles, described in 1948 by Arnold Kegel. On the other hand, women may have difficulties in identifying and controlling this group of muscles^(17)^. Plevnik^(6)^ developed VCs to solve this dilemma. According to the Cochrane database in 2013, there was a statistically significant difference between a VC and no treatment group regarding pelvic muscle strength and pad test. In this review, cones had no additional benefit over PFMT and electrostimulation therapy^(4)^. In our study, we compared the effectiveness of VC therapy with TOT. To the best of our knowledge, this is the first randomized study to compare surgical and conservative treatment of SUI. In our trial, all women were multiparous (the rate of parities are 3.5 for TOT and 3.1 for VC) and all women’s body mass indexs were over 30 kg/m^2^; the SUI for both groups showed a strong association with the parity and obesity (Table 1)^(18,19)^. The duration of TOT procedures (14±2 minutes) and intraoperative median blood loss (35 mL) were compatible with previous studies^(10,20,21)^. There were no intraoperative complications in our study. VCs are treatment options that have some advantages including the ease of learning how to implement them, which reduces patient visits, and ease of use at home^(22)^. In our study, treatment adherence was high with a reported integration of 100% of the participants. At the 6^th^ week and 6^th^ month follow ups, slightly varying subjective cure rates were observed between the two groups (group 1, 65% vs. group 2, 90%) (group 1, 75% vs. group 2, 80%), respectively, but there were no significant differences in their subjective cure rates (p>0.05). However, the objective cure rates were significantly different between the two groups at the 6^th^ week (VC, vs. 10% vs. TOT, 80%) and 6^th^ month follow ups (VC 30% vs. TOT 75%). After the 6 weeks and also 6 months of follow up, the rates were statistically different in the two groups (p<0.05). All patients were analyzed using a quality of life questionnaire before and after treatment. Although the subjective cure rates were similar, the objective cure rate was higher in the TOT group, as expected (p<0.05). Our study is the first randomized control study to compare TOT and VC treatment; however, it is not without limitations. One of the main limitations of this study is the limited number of patients in the study group. Also, we were not be able to compare various VC weights. In our study, we used fixed weights (20-70 g), which may have affected the subjective and objective cure rates.

## Conclusion

TOT is highly efficient minimally invasive surgical procedure with high success rates and low complication rates. VC should not be considered as one of the alternatives to surgical treatment. VCs are cheap and simple conservative methods. VCs may be considered temporarily or in combination with other surgical procedures. Cones could be offered as a treatment option for women who are scheduled for surgery or in those at high risk for surgery if they find them acceptable.

## Figures and Tables

**Table 1:**
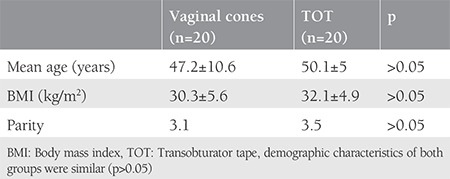
Clinical and demographic characteristics of study groups

**Table 2:**
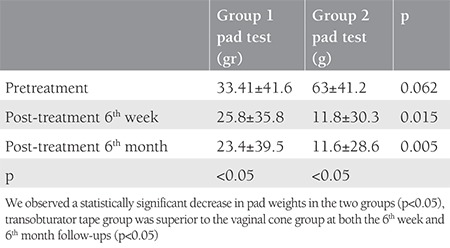
Outcome measure and multiple comparisons of all groups at 6 weeks and 6 months

**Table 3:**
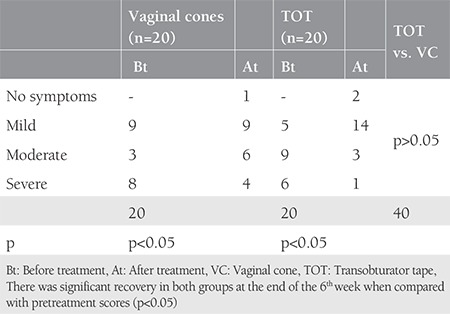
The results of quality of life questionnaire in all groups at 6^th^ weeks

**Table 4:**
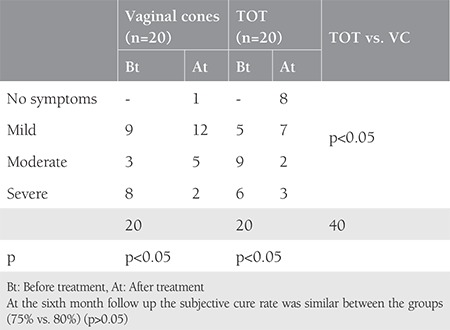
The results of quality of life questionnaire in all groups at 6 months
